# Supplementary material for: Dynamic earthquake rupture preserved in a creeping serpentinite shear zone
Source: Nat Commun. 2018 Sep 3;9:3552. doi: 10.1038/s41467-018-05965-0 (PMC6120932; doi:10.1038/s41467-018-05965-0)
Supplement: Supplementary file 1 — Supplementary Information [file 41467_2018_5965_MOESM1_ESM.docx]

Supplementary Information


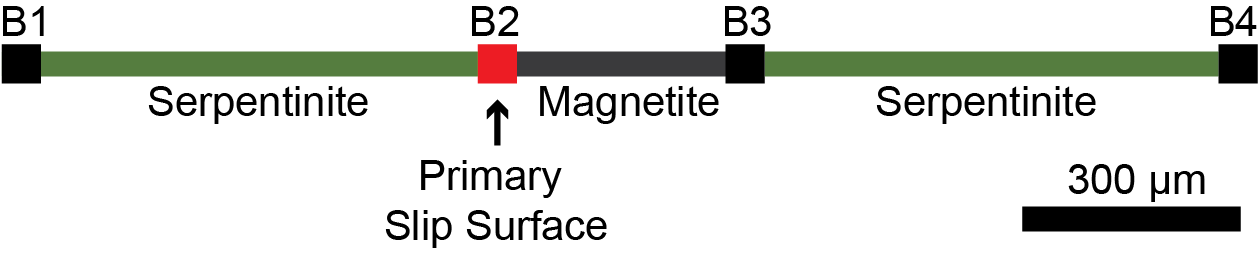


Supplementary Figure 1: Schematic of the 1D model for coseismic frictional heating.


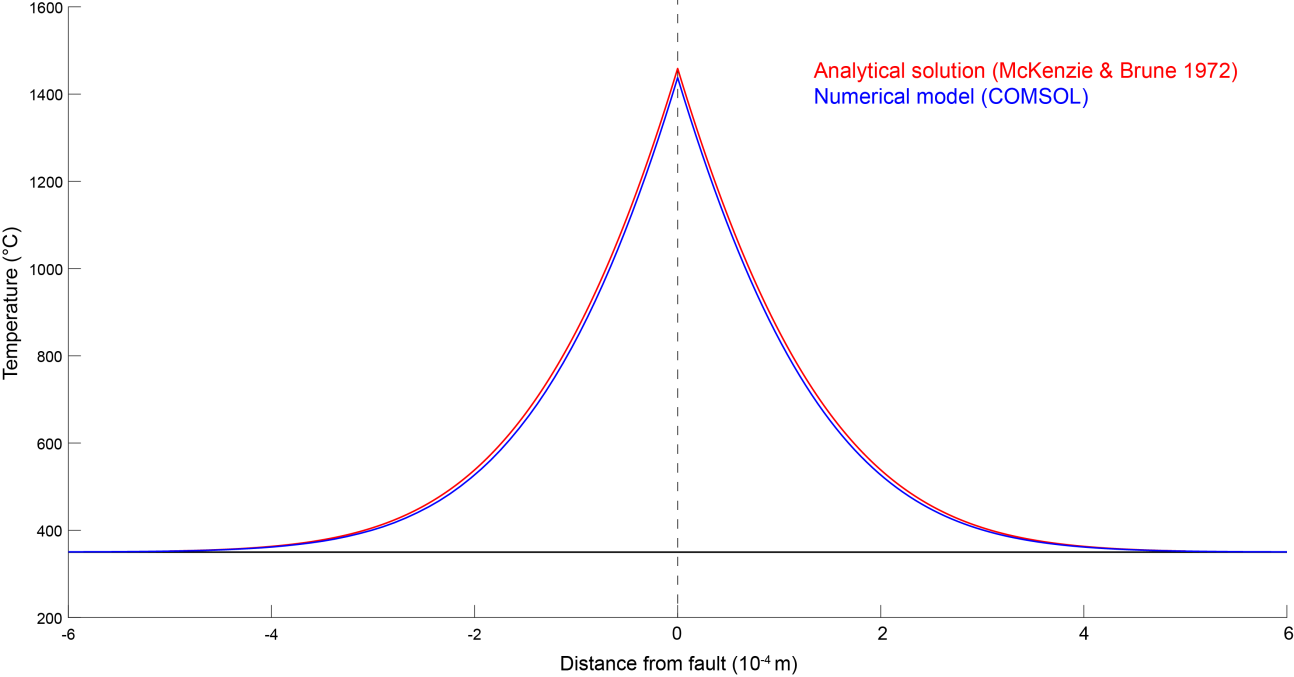


Supplementary Figure 2: Comparison between results of the analytical solution (McKenzie & Brune 1972) and the numerical model.

| Reaction observed | Onset of serpentine dehydration | Formation of talc | Formation of nanocrystalline forsterite | Disappearance of talc | Development of well-crystallised forsterite | Formation of enstatite |
| --- | --- | --- | --- | --- | --- | --- |
| Mineral Assemblage | e. Partially amorphous serpentine | f. Partially amorphous serpentine and talc | g. Poorly crystalline olivine and talc | h-i. Moderately-crystalline olivine | j-k. Well-crystallised olivine aggregates | l. Crystalline olivine and enstatite |
| ref^1^ | 550 | - | 750 | - | - | - |
| ref ^2^ | - | - | - | 800 | - | - |
| ref ^2^ | - | - | - | 830 | - | - |
| ref ^3^ | 580 | 613 | 639 | 768 | 871 | - |
| ref ^4^ | 600 | - | 650 | - | 850 | 1000 |
| ref ^5^ | 450 | 665 | 639 | 768 | - | - |
| ref ^6^ | 550 | - | 650 | 900 | - | 800 |
| ref ^7^ | 600 | - | 820 | - | - | 830 |
| ref ^8^ | 500 | - | - | - | - | 824 |
| ref ^9^ | 550 | `- | 775 | - | 875 | 820 |
| ref ^10^ | 600 | 587 | 600 | 700 | 800 | 800 |
| ref ^11^ | 550 | - | 700 | - |  | 800 |
| ref ^6^ | 500 | - | - | - | 1100 | 1100 |
| ref ^12^ | 600 | - | 670 | - | - | 1150 |
| ref ^13^ | 580 | - | 640 | - | 750 | 900 |
| ref ^14^ | 620 | - | 790 | - | - | 1135 |
| ref ^15^ | 600 | - | 810 | - | - | 1000 |
| ref ^16^ | 580 | - | 620 | 810 | - | 800 |
| ref ^17^ | - | - | - | 850 | - |  |
| ref ^18^ | - | - | 600 | - | 800 | 1000 |
| ref ^19^ | 659 | - | - | - | - | - |
| ref ^20^ | - | - | 500 | - | - | 1000 |
| Average | 569 | 622 | 695 | 803 | 833 | 926 |
| Standard error | 25 | 45 | 46 | 42 | 84 | 62 |

**Supplementary Table 1**: Compilation of data from thermal analysis experiments on serpentine (lizardite and/or chrysotile). Temperatures (°C) correspond to first appearance or disappearance of the dehydration product.

| Parameter | Symbol | Value | Units |
| --- | --- | --- | --- |
| Coefficient of friction | $\mu$ | 0.4 ^21^ |  |
| Slip velocity | $v$ | 1 | m s^-1^ |
| Density of serpentinite | $\rho_{serpentinite}$ | 2600 | kg m^-3^ |
| Density of water | $\rho_{w}$ | ^22^ | kg m^-3^ |
| Density of forsterite | $\rho_{sforsterite}$ | 3200 | kg m^-3^ |
| Density of magnetite | $\rho_{magnetite}$ | 5200 | kg m^-3^ |
| Normal stress | $\sigma_{N}$ | 270 | MPa |
| Pore fluid pressure (initial) | $p_{0}$ | 0.3$\sigma_{N}$ | MPa |
| Initial temperature | $T_{0}$ | 350 | °C |
| Heat capacity serpentinite | ${C_{p}}_{serpentinite}$ | ref^23^ | J K^-1^ |
| Heat capacity magnetite | ${C_{p}}_{magnetite}$ | ref^24^ | J K^-1^ |
| Heat capacity forsterite | ${C_{p}}_{forsterite}$ | ref^25,26^ | J K^-1^ |
| Thermal conductivity serpentinite | $k_{serpentinite}$ | ref^27^ | W m^-1^ K^-1^ |
| Thermal conductivity magnetite | $k_{magnetite}$ | ref^24,28^ | W m^-1^ K^-1^ |
| Thermal conductivity forsterite | $k_{forsterite}$ | ref^25,26^ | W m^-1^ K^-1^ |
| Enthalpy heat of serp. dehydration | $Q_{d}$ | 521 ^29^ | kJ mol^-1^ |
| Thermal expansion of water | $\lambda_{n}$ | ref^22^ | K^-1^ |
| Thermal expansion of pores | $\lambda_{f}$ | 0.02 ^30^ | 10^−3^ K^-1^ |
| Compressibility of pores | $\beta_{n}$ | 2.49 ^30^ | 10^−9^ Pa^-1^ |
| Compressibility of water | $\beta_{f}$ | ref^22^ | Pa^-1^ |
| Porosity | $n$ | 0.01 |  |
| Reaction induced porosity change | $\Delta n$ | 0.24 ^31^ |  |
| Viscosity of water | $\eta_{w}$ | ref^32^ | Pa s |
| Permeability of serpentinite | $K_{s}$ | 10^-19 31^ | m^2^ |
| Moles of water released ^a^ | $\nu_{s}$ | $\frac{9}{5}$ |  |
| Mass fraction of serpentine ^b^ | $w_{serpentine}$ | 0.85 |  |
| Molar mass of water | $M_{H_{2}O}$ | 18.0 | g/mol |
| Molar mass of serpentinite | $M_{serpentine}$ | 277.1 | g/mol |

^a^ moles of water released per mol of serpentine in dehydration reaction (1) in the main text.

^b^ mass fraction of hydrous serpentine in the dehydrating serpentinite. Remaining mass is accounted for by magnetite and minor relict spinels.

Supplementary Table 2: Values used in the numerical model. Parameters without specific values are temperature dependent and references are provided that include information on the temperature- dependence.

**Supplementary References**

1. Cao, C. Y., Liang, C. H., Yin, Y. & Du, L. Y. Thermal activation of serpentine for adsorption of cadmium. *J. Hazard. Mater.* **329,** 222–229 (2017).

2. Wang, D., Yi, L., Huang, B. & Liu, C. High-temperature dehydration of talc: a kinetics study using in situ X-ray powder diffraction. *Phase Transitions* **88,** 560–566 (2015).

3. Trittschack, R. & Grobéty, B. The dehydroxylation of chrysotile: A combined in situ micro-Raman and micro-FTIR study. *Am. Mineral.* **98,** 1133–1145 (2013).

4. Zulumyan, N., Mirgorodski, A., Isahakyan, A. & Beglaryan, H. The mechanism of decomposition of serpentines from peridotites on heating. *J. Therm. Anal. Calorim.* **115,** 1003–1012 (2014).

5. Trittschack, R., Grobéty, B. & Koch-Müller, M. The lizardite phase transformation followed by in situ high-temperature Raman and FTIR spectroscopy. *Am. Mineral.* **97,** 1965–1976 (2012).

6. Gualtieri, A. F., Giacobbe, C. & Viti, C. The dehydroxylation of serpentine group minerals. *Am. Mineral.* **97,** 666–680 (2012).

7. Kusiorowski, R., Zaremba, T., Piotrowski, J. & Adamek, J. Thermal decomposition of different types of asbestos. *J. Therm. Anal. Calorim.* **109,** 693–704 (2012).

8. Viti, C., Giacobbe, C. & Gualtieri, A. F. Quantitative determination of chrysotile in massive serpentinites using DTA: Implications for asbestos determinations. *Am. Mineral.* **96,** 1003–1011 (2011).

9. Viti, C. Serpentine minerals discrimination by thermal analysis. *Am. Mineral.* **95,** 631–638 (2010).

10. Candela, P. A., Crummett, C. D., Earnest, D. J., Frank, M. R. & Wylie, A. G. Low-pressure decomposition of chrysotile as a function of time and temperature. *Am. Mineral.* **92,** 1704–1713 (2007).

11. Cattaneo, A., Gualtieri, A. F. & Artioli, G. Kinetic study of the dehydroxylation of chrysotile asbestos with temperature by in situ XRPD. *Phys. Chem. Miner.* **30,** 177–183 (2003).

12. Mackenzie, K. J. D. & Meinhold, R. H. Thermal reactions of chrysotile revisited: a 29Si and 25Mg MAS NMR study. *Am. Mineral.* **79,** 43–50 (1994).

13. Datta, A. K. Dehydration of chrysotile asbestos: an infrared absorption study. *J. Mater. Sci. Lett.* **10,** 870–871 (1991).

14. Ashimov, U. B., Bolotov, Y. A., Arykbaev, R. K. & Shipkov, N. V. Thermal analysis of serpentinites. *Refractories* **30,** 491–494 (1989).

15. Jolicoeur, C. & Duchesne, D. Infrared and thermogravimetric studies of the thermal degradation of chrysotile asbestos fibers: evidence for matrix effects. *Can. J. Chem.* **59,** 1521–1526 (2011).

16. Martin, C. J. The Thermal Decomposition of Chrysotile. *Mineral. Mag.* **41,** 453–459 (1977).

17. Richard Ward, J. Kinetics of talc dehydroxylation. *Thermochim. Acta* **13,** 7–14 (1975).

18. Brindley, G. W. & Hayami, R. Mechanism of formation of forsterite and enstatite from serpentine. *Mineral. Mag.* **35,** 189–195 (1965).

19. Weber, J. N. & Greer, R. T. Dehydration of Serpentine: Heat of Reaction and Reaction Kinetics At P_H2O_ = 1 Atm. *Am. Mineral.* **50,** 450–464 (1965).

20. Ball, M. C. & Taylor, H. F. W. The dehydration of chrysotile in air and under hydrothermal conditions. *Mineral. Mag.* **33,** 467–482 (1963).

21. Brantut, N., Schubnel, A., Corvisier, J. & Sarout, J. Thermochemical pressurization of faults during coseismic slip. *J. Geophys. Res. Solid Earth* **115,** 1–17 (2010).

22. Wagner, W. & Pruß, A. The IAPWS formulation 1995 for the thermodynamic properties of ordinary water substance for general and scientific use. *J. Phys. Chem. Ref. data* **31,** 387–535 (2002).

23. Osako, M. *et al.* Thermal diffusivity, thermal conductivity and heat capacity of serpentine (antigorite) under high pressure. *Phys. Earth Planet. Inter.* **183,** 229–233 (2010).

24. Grosu, Y., Faik, A., Ortega-Fernández, I. & D’Aguanno, B. Natural Magnetite for thermal energy storage: Excellent thermophysical properties, reversible latent heat transition and controlled thermal conductivity. *Sol. Energy Mater. Sol. Cells* **161,** 170–176 (2017).

25. Xu, Y. *et al.* Thermal diffusivity and conductivity of olivine, wadsleyite and ringwoodite to 20 GPa and 1373 K. *Phys. Earth Planet. Inter.* **143,** 321–336 (2004).

26. Cynn, H., Carnes, J. D. & Anderson, O. L. Thermal properties of forsterite, including C_V_, calculated from αK_T_ through the entropy. *J. Phys. Chem. Solids* **57,** 1593–1599 (1996).

27. Seipold, U. & Schilling, F. R. Heat transport in serpentinites. *Tectonophysics* **370,** 147–162 (2003).

28. Levy, D., Giustetto, R. & Hoser, A. Structure of magnetite (Fe_3_O_4_) above the Curie temperature: A cation ordering study. *Phys. Chem. Miner.* **39,** 169–176 (2012).

29. Llana-Fúnez, S., Brodie, K. H., Rutter, E. H. & Arkwright, J. C. Experimental dehydration kinetics of serpentinite using pore volumometry. *J. Metamorph. Geol.* **25,** 423–438 (2007).

30. Rice, J. R. Heating and weakening of faults during earthquake slip. *J. Geophys. Res. Solid Earth* **111,** 1–29 (2006).

31. Tenthorey, E. & Cox, S. F. Reaction-enhanced permeability during serpentinite dehydration. *Geology* **31,** 921–924 (2003).

32. Huber, M. L. *et al.* New international formulation for the viscosity of H_2_O. *J. Phys. Chem. Ref. Data* **38,** 101–125 (2009).
